# Evolutionary Spiking Neural Networks for Solving Supervised Classification Problems

**DOI:** 10.1155/2019/4182639

**Published:** 2019-03-28

**Authors:** G. López-Vázquez, M. Ornelas-Rodriguez, A. Espinal, J. A. Soria-Alcaraz, A. Rojas-Domínguez, H. J. Puga-Soberanes, J. M. Carpio, H. Rostro-Gonzalez

**Affiliations:** ^1^Postgraduate Studies and Research Division, National Technology of Mexico, León Institute of Technology, León, Guanajuato, Mexico; ^2^Department of Organizational Studies, DCEA-University of Guanajuato, Guanajuato, Guanajuato, Mexico; ^3^Department of Electronics, DICIS-University of Guanajuato, Salamanca, Guanajuato, Mexico

## Abstract

This paper presents a grammatical evolution (GE)-based methodology to automatically design third generation artificial neural networks (ANNs), also known as spiking neural networks (SNNs), for solving supervised classification problems. The proposal performs the SNN design by exploring the search space of three-layered feedforward topologies with configured synaptic connections (weights and delays) so that no explicit training is carried out. Besides, the designed SNNs have partial connections between input and hidden layers which may contribute to avoid redundancies and reduce the dimensionality of input feature vectors. The proposal was tested on several well-known benchmark datasets from the UCI repository and statistically compared against a similar design methodology for second generation ANNs and an adapted version of that methodology for SNNs; also, the results of the two methodologies and the proposed one were improved by changing the fitness function in the design process. The proposed methodology shows competitive and consistent results, and the statistical tests support the conclusion that the designs produced by the proposal perform better than those produced by other methodologies.

## 1. Introduction

Artificial neural networks (ANNs) have been successfully used in theoretical and practical fields to solve several kinds of problems (e.g., classification [1, 2], robotic locomotion [3, 4], and function approximation [5, 6]). Basically, ANNs are characterized by *computing units* which are interconnected through *communication links* that serve to send and/or receive *messages of some data type* [7]; these elements define what is known as their architecture or topology. There can be distinguished three generations of ANNs according to their computing units [8], which are capable to solve problems of digital (ANNs from 1st to 3rd generation), analogical (ANNs from 2nd to 3rd generation), and spatiotemporal (ANNs from 3rd generation) nature. The first generation is based on threshold units such as McCulloch–Pitts neurons [9] or perceptrons [10]. The second generation is based on computing units that apply continuous activation functions (e.g., sigmoid or hyperbolic tangent functions); ANNs of this generation can be trained with gradient descent-based algorithms such as the backpropagation learning rule [11]. The third generation is based on spiking neurons (see [12] for a detailed reference) such as integrate and fire model [13] or Hodgkin–Huxley neuron [14]; ANNs of this generation are known as spiking neural networks (SNNs), and these are the kinds of ANNs worked in this paper.

Usually, the implementation of an ANN to solve a specific problem, regardless the generation it belongs to, requires of human experts who define the ANN's topological elements, the learning rule, and its parameters, among other design criteria. The experts perform such a design process either empirically, following some rule of thumb or by trial and error; this is due because there is a lack of a well-established methodology to set up the ANN design for a given problem. It is well-known that the good performance of ANNs is strongly related to their design and related criteria; thus, design of an ANN may stand for a challenge. Several studies have explored the learnability issues of ANNs related to their design; for example, combinatorial problems that arise related to the design of feedforward ANNs [15] or the problems that ANNs with fixed architectures may face when learning a specific problem [7, 16–19]. Insights have been given to ease or enhance learnability of ANNs, for example, by applying constraints to the task to be learned or to the ANN's architecture [7, 15]. As an example of constraints applied to the ANNs' architecture, partially connected ANNs have shown equal or better performance than their fully connected version; among other interesting benefits, there are reduction of the network complexity and its training and recall times [20]. Another insight is to develop algorithms capable of changing the architecture of an ANN during the learning process [7, 15].

Nowadays, evolutionary artificial neural networks (EANNs) are a special class of ANNs which are the result of using evolutionary algorithms (EAs), or other kinds of metaheuristic methods, for adapting the design of ANNs according to a specific task or problem; this is achieved by optimizing one or several of their design criteria (also the term *Neuroevolution* has been used to refer to this kind of design method). Thus, the EANNs, in some manner, allow us to avoid or overcome the learnability issues related to ANN architectures and to prescind, partially or completely, of human experts (see [21–24] for comprehensive reviews). There are four main approaches of deploying EANNs [25] by means of weight optimization [26–28], topology structure optimization [25, 29–31], weight and topology structure optimization [32–38], and learning rule optimization [39, 40]. Most of the work made on EANNs is focused on deploying ANNs from the first and second generations.

Recently, efforts to use SNNs for solving real problems from engineering and industry are increasing because of interesting characteristics of spiking neurons, such as their greater computational power than that of less plausible neuron models and SNNs can solve problems with fewer computing units than those of ANNs from previous generations [19, 41]. Although there are learning rules to adapt parameters of SNNs, such as SpikeProp [42], the use of metaheurstic algorithms is a common practice to adapt their parameters or define design criteria because they overcome drawbacks of such learning rules [43] and allow us to handle the greater variety of design criteria (parameters of neuron models and synapses, types of synapses, topology's wiring patterns, encoding scheme, etc.) that these kinds of ANNs present; in this work, the combination of SNN and metaheuristic algorithms is referred as evolutionary spiking neural networks (ESNNs). In [44–48], the synaptic weights of a single spiking neuron, e.g., integrate and fire model [13] or Izhikevich model [49], are calibrated by means of algorithms such as differential evolution (DE) [50], particle swarm optimization (PSO) [51], cuckoo search algorithm (CSA) [52], or genetic algorithm (GA) [53] to perform classification tasks; the spiking neuron performs the classification by using the firing rate encoding scheme as the similarity criterion in order to assign the class to which an input pattern belongs. Other works, in [43, 54–57], three-layered feedforward SNNs with synaptic connections were implemented, which are formed by a weight and a delay, to solve supervised classification problems through the use of time-to-first-spike as a classification criterion; in these works, the training has been carried out by means of evolutionary strategy (ES) [58, 59] and PSO algorithms. An extension of previous works is made in [60, 61], where the number of hidden layers and their computing units are defined by grammatical evolution (GE) [62] besides the metaheuristic learning. More complex SNN frameworks have been developed and trained with metaheuristics (such as ES) to perform tasks such as visual pattern recognition, audio-visual pattern recognition, taste recognition, ecological modelling, sign language recognition, object movement recognition, and EEG spatio/spectrotemporal pattern recognition (see [63] for a review of these frameworks). The robotic locomotion is solved through SNNs designed by metaheuristics in [60, 64, 65]; in these works, both the connectivity pattern and synaptic weights of each Belson–Mazet–Soula (BMS) [66] neuron model into SNNs called spiking central pattern generators (SCPGs) are defined through GE or Christiansen grammar evolution (CGE) [67] algorithms; all individual designs are integrated to define the SCPGs that allow the locomotion of legged robots.

The present paper proposes a design methodology for three-layered feedforward ANNs of the third generation for solving supervised classification problems. The design methodology incorporates partial connectivity between input and hidden layers, which contribute to reduce the topological complexity of the ESNNs; in addition, partial connectivity may also contribute to reduce the number of features of the input vector, thus indirectly performing dimensionality reduction. The proposal explores the search space of three-layered feedforward topologies with configured synaptic connections; thus an explicit learning process is not required. This kind of design methodology has been previously proposed for ANNs from first and second generations, and they can be considered as a design of composed functions. To the best of the authors' knowledge, this is the first attempt to perform the design of SNNs that define the number of computing units and their configured connectivity patterns (weights and delays). The rest of the paper is organized as follows: Section 2 explains the proposed methodology and its constituent methods. The experimental configuration of the proposal and other methodologies used for comparison and their results are in Section 3. In Section 4, the results of the proposed methodology are statistically compared to those of other methodologies. Finally, Section 5 contains the conclusion of the paper and future work based on it.

## 2. Design Methodology and Concepts

**Figure 1:**
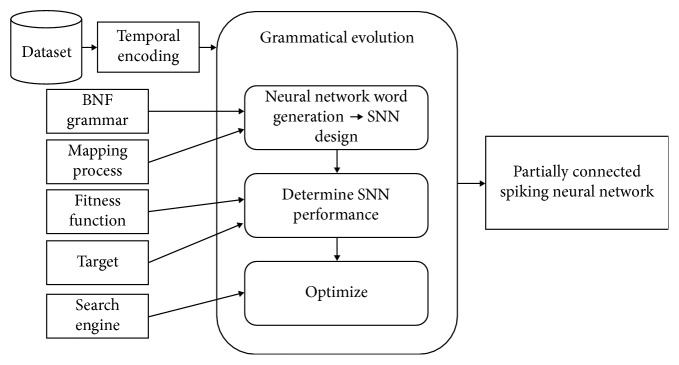
General diagram for the proposed framework.

This paper proposes a framework to design partially connected spiking neural networks (SNNs) for solving supervised classification problems. Such proposed framework requires the following elements: a *temporal encoding* scheme to transform original input data into a suitable form for the network; a context-free grammar in Backus–Naur form (BNF grammar) to guide the generation of neural network words and a *mapping process* to transform genotype of individuals into functional network designs; a *fitness function* and a *target* definition to determine the performance of proposed networks, and a *search engine* to optimize the solutions. A general diagram of the methodology can be seen in Figure 1.

### 2.1. Spiking Neural Networks

The spiking neural networks (*SNNs*) constitute the third generation of *ANNs* because of the inclusion of the firing time component in their computation process [8].

#### 2.1.1. Spike Response Model

**Figure 2:**
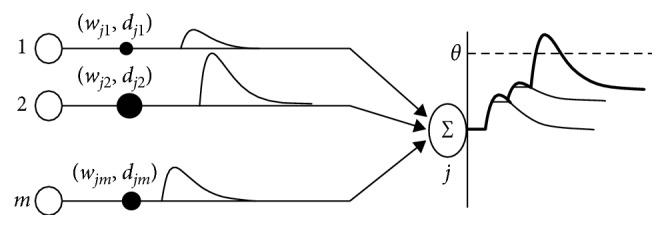
Membrane potential of neuron *j*: linear summation of PSPs [55].

The spike response model (SRM) is employed in this framework as basis for the SNN. The SRM fires (i.e., produces a spike) whenever the state of its membrane potential surpasses the firing threshold (*θ*). In the SRM, its membrane potential is calculated through time as a linear summation of postsynaptic potentials (PSPs) (excitatories and/or inhibitories), which are caused by impinging spikes arriving to a neuron through its presynaptic connections (Figure 2); each PSP is weighted and delayed by its synaptic connection.

The membrane potential *x* of neuron *j* at time *t* is calculated as the weighted (*w*_*ji*_) summation of contributions (*y*_*i*_(*t*)) from its connecting presynapses (Γ_*j*_), as in the following equation:

(1) $$\begin{array}{c} x_{j}\left(t\right)=\underset{i\in \Gamma_{j}}{\sum }w_{ji}y_{i}\left(t\right). \end{array}$$

The unweighted contribution *y*_*i*_(*t*) is described by equation (2), in which the function *ε*(*t* − *t*_*i*_ − *d*_*ji*_) describes a form of the PSPs generated by impinging spikes coming from the presynaptic neuron *i* at the simulation time (*t*). The parameters of the presynaptic connection *i* are: the firing time *t*_*i*_ and synaptic delay *d*_*ji*_:

(2) $$\begin{array}{c} y_{i}\left(t\right)=\varepsilon \left(t-t_{i}-d_{ji}\right). \end{array}$$

The spike response function *ε*(*t*) describes the form of PSPs, and it is defined in the following equation, where *τ* represents the membrane potential time constant that defines the decay time of the postsynaptic potential:

(3) $$\begin{array}{c} \varepsilon \left(t\right)=\left\{\begin{array}{cc} \frac{t}{\tau }e^{1-t/\tau }, & \text{if}\;\;t>0,\\ 0, & \text{else}. \end{array}\right. \end{array}$$

### 2.2. Temporal Encoding

Due to the nature of the employed neural model, original features from the dataset must be transformed into spikes prior to introducing them into the network. For such purpose, the one-dimensional encoding in the following equation is employed [56]:

(4) $$\begin{array}{c} Y\left(f\right)=\left[\frac{\left(b-a\right)}{r}*f\right]+\left[\frac{\left(a*M\right)-\left(b*m\right)}{r}\right], \end{array}$$

where *Y* is the spike temporal value, *f* is the original feature value, [*a*, *b*] are the lower and upper temporal interval limits of the encoding, whereas *M* and *m* hold the maximum and minimum values that the *f* variable takes, respectively, and *r* is the range between *M* and *m*. This encoding method preserves the dimension of the samples in the dataset, while providing a temporal representation of the scalar values of the dataset suitable for insertion in the network.

### 2.3. Grammatical Evolution (GE)

Grammatical evolution is an evolutionary algorithm based on the combination of genetic algorithms and context-free grammars [68]. It employs a BNF grammar relating to the problem, a mapping process to obtain the functional form of solutions, and a search engine to drive the search process.

#### 2.3.1. BNF Grammar

**Figure 3:**
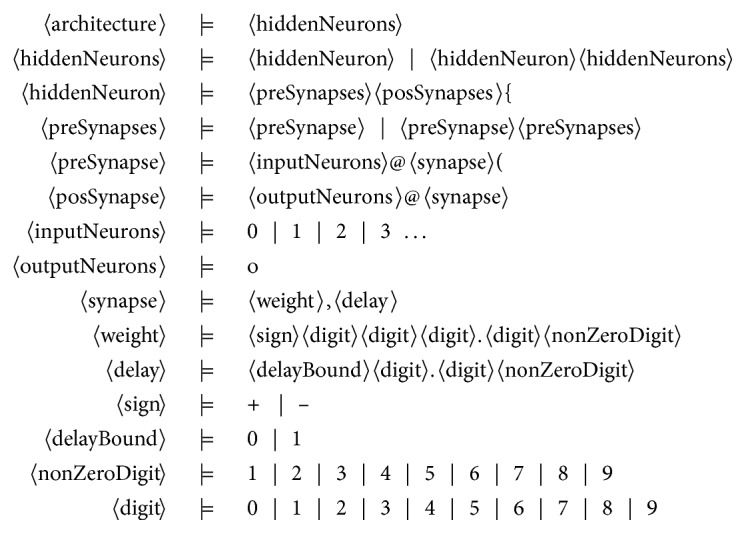
Proposed BNF grammar for designing partially connected SNNs.

The *Backus–Naur form* (Figure 3) is employed to define the topology of the network and its parameters. Any word produced by this grammar includes an arbitrary number of hidden neurons and some specific *pre-* and *post*synapses with their respective parameters. The opening curled bracket symbol ({) indicates the division between hidden neurons, while the opening parenthesis (() marks the different synapses, and the *at* symbol (@) precedes the synapse-specific weight and delay values.

**Figure 4:**
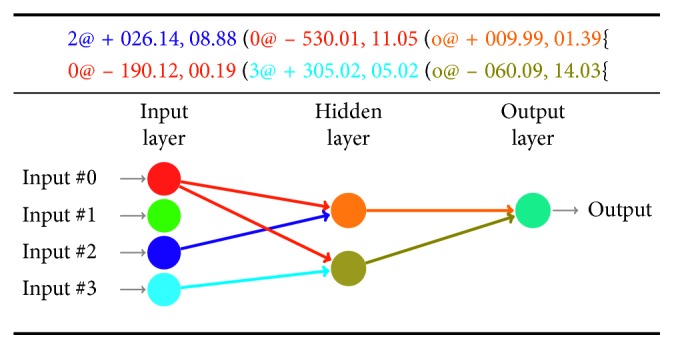
Example of a word generated by the proposed grammar and its corresponding network topology.

Figure 4 illustrates an example of a word generated by the proposed grammar and its corresponding network topology. By relating the word with its network topology, the word has two “{” symbols (see end of each row), implying that the network has two hidden neurons. In this case, each row has two “(” symbols meaning three synaptic configurations (but it can vary for each hidden neuron), where the first and second synaptic configurations represent connections with neurons from the input layer, and the last configuration marks the synapse with the output layer; each synaptic configuration is formed by a neuron identifier, a synaptic weight, and a delay. In Figure 4, each presynaptic neuron and its synaptic connection with a postsynaptic neuron are portrayed in the same color to clarify the reading of the transformation process from a word to a network topology.

#### 2.3.2. Mapping Process

The mapping process transforms an individual from its genotypic form into its phenotypic form to represent a functional network. The depth-first mapping process—employed in this framework—is the standard in GE; basically, it begins by deriving (i.e., replace it by one of its productions) the left-most nonterminal symbol (initially, <*architecture>* non-terminal symbol) until all nonterminal symbols in depth are derived and then moves to the current left-most nonterminal. The process continues until either nonterminals are depleted, or all elements of the genotype have been used.

#### 2.3.3. Search Engine

Several population-based metaheuristic algorithms can be used as the search engine of grammatical evolution. The well-known genetic algorithm (GA) and differential evolution (DE) are used in this framework [69].

### 2.4. Fitness Function

Two different fitness functions are considered to provide a measure of the ability of the solutions to solve the problem:

1. The squared error is as defined in the following equation, where *P* is the total number of training patterns, *O* is the number of neurons in the output layer, *t*_*o*_^*a*^(*p*) is the actual firing time, and *t*_*o*_^*d*^(*p*) is the desired firing time of neuron *o*:

(5) $$\begin{array}{c} E_{s}=\overset{P}{\underset{p}{\sum }}\overset{O}{\underset{o}{\sum }}{\left(t_{o}^{a}\left(p\right)-t_{o}^{d}\left(p\right)\right)}^{2}. \end{array}$$

(2) The accuracy error of the training subset is as in the following equation, where *C* is the number of correct predictions and *T* is the total of predictions:

(6) $$\begin{array}{c} E_{a}=1-\left(\frac{C}{T}\right). \end{array}$$

Both fitness functions are designed to be minimized.

### 2.5. Target

In order to obtain a prediction, a particular firing time is assigned to each class in the dataset employed, resulting in a desired time-to-first spike for every sample belonging to a specific class.

## 3. Experiments and Results

Twelve supervised classification benchmark datasets from the UCI Machine Learning Repository [70] were considered for experimentation: Balance Scale, Blood Transfusion Service Center (Blood), Breast Cancer Wisconsin (Breast Cancer), Japanese Credit Screening (Card), Pima Indians Diabetes (Diabetes), Fertility, Glass Identification (Glass), Ionosphere, Iris Plant, Liver Disorders (Liver), Parkinson, and Wine.  Table 1 shows the details of the datasets employed.

Each dataset was randomly divided into two subsets of approximately the same size, accounting for the instances of each class to be evenly distributed between the subsets. One of these subsets is assigned to be the design set, while the other is to be the test set.

Then, the design set is employed to carry out the GE, while the test set is reserved to prove the performance of the best solution provided by the evolutionary process.

Aiming to compare the performance between neural models from different generations in solving pattern recognition tasks, six different configurations were considered, as shown in Table 2, observing the following details:

- *α* configurations employ the parameters defined in [35], focusing on developing second-generation partially connected ANNs
- *β* configurations aim to be an homology of *α* configurations but used to produce third-generation partially connected ANNs
- *γ* configurations are defined as *β* configurations but employing DE as search engine instead of GA

Parameters between configurations were matched to make a comparison as fair as possible. Furthermore, configurations labeled with subscript 1 look upon the squared error as the fitness function to guide the evolutionary process, while configurations labeled with subscript 2 consider the accuracy error of the design set.

In order to guarantee statistical significance, the central limit theorem [71] is satisfied by performing 33 experiments for each configuration. Specific parameters used in this framework for configurations *β* and *γ* are provided next.

  Temporal Encoding: The one-dimensional encoding scheme observes a temporal range from 0.01 to 9 milliseconds (ms).
  SRM: *membrane potential time constant τ* = 9; target: {12 ms, 15 ms, 18 ms, … .} (depending on the number of classes in the dataset); *simulation time* [10 ms, target of the last class plus two]; *threshold θ* = 1 millivolts (mV); *weight range* ∈ [−999.99, 999.99]; and *delay range* ∈ [0.01, 19.99] (ms).
  GA: binary search space [0, 1], *codon size* = 8; *individual dimension* = 4000 (500 codons); *population size* = 100; *function calls* = 1,000,000; *K*-tournament (*K* = 5) selection operator; *elitism percentage* = 10%; one-point crossover operator; *mutation:* bit-negator mutation operator (5%).
  DE: real search space [0, 255]; *individual dimension* = 500; *function calls* = 1,000,000; *population size* = 100; *crossover rate* = 10%; *mutation*: DE/Rand/1.

Tables 3 and 4 show the results obtained by carrying out the aforementioned methodology. Accuracy value ∈ [0, 1] grades the average performance of the configurations applied to classify specific datasets, along with its corresponding standard deviation, for all experiments made. Design accuracy relates with the performance of the best network topology obtained by the evolutionary algorithm, whilst test accuracy indicates the performance of such network applied to the test subset; highest values are indicated in boldface.

As well, Tables 5 and 6 show some of the features of the generated topologies, focusing on the average amount of input vector features actually employed by the networks, and its corresponding rate regarding the total size of the original input vector; besides, the average number of hidden units and synapses present in the generated networks. In Supplementary Materials, some examples of SNNs' topologies with best obtained results are shown; each example contains the benchmark dataset, used configuration, accuracies of design and test phases, the generated word, and the network topology.

## 4. Comparative Statistical Analysis

As detailed in the previous section, data samples from performing thirty-three independent experiments for each configuration on every dataset were obtained. Thereupon, several statistical tests [72] were applied to these data. First of all, a Shapiro–Wilk [73] test was applied to determine the normality of the samples. Such test showed that data can indeed be modelled under normal distributions. Further analysis was divided into three tests applied to configurations using squared error as fitness function, configurations using accuracy error as fitness function, and all configurations.

### 4.1. Test of Designs Driven by Squared Error Fitness Function

In order to verify statistical significance of the results, analysis of variance (ANOVA [74]) tests were applied to determine if, firstly, implementing different methodologies to develop weighed network topologies impacts on the accuracy of classification and secondly, to identify which of these methodologies offers the best performance.  Table 7 shows the results obtained by two-way ANOVA test, observing as independent variables both configurations and datasets.

ANOVA's null hypothesis (*H*_0_) dictates that observed samples come from one unique normal distribution. As *p* values (Pr(*>F*) in Table 7) are smaller than the significance value of 0.05, there is not enough evidence to accept *H*_0_, ergo rejecting that samples are statistically similar. In other words, it can be conclude that configurations come from different distributions. This test provides relevant statistical evidence to support the conclusion that changing the methodology while generating weighted topologies influences the classification accuracy of the networks.

**Figure 5:**
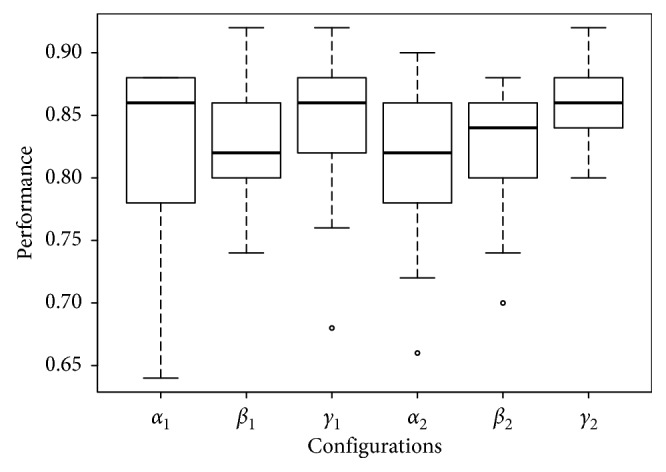
Box plots of the performance of all configurations on the Fertility dataset.

**Figure 6:**
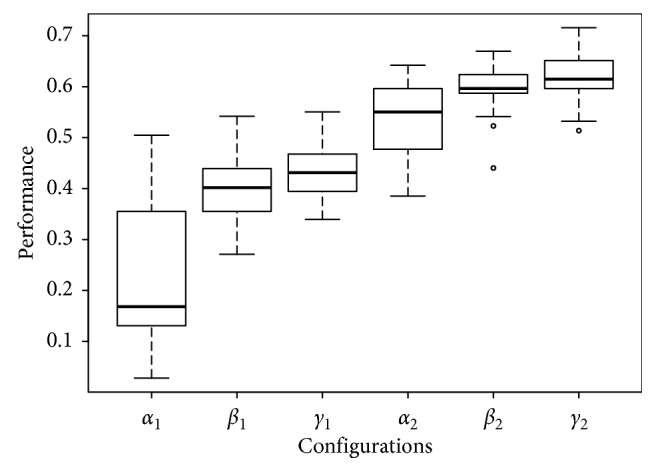
Box plots of the performance of all configurations on the Glass dataset.

**Figure 7:**
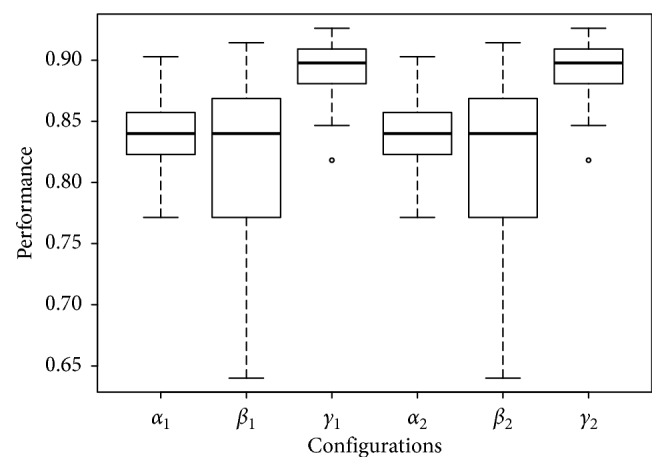
Box plots of the performance of all configurations on the Ionosphere dataset.

Pairwise *t*-tests and *Tukey HSD* [75] tests were applied next. As in the ANOVA test, the null hypothesis in both tests assumes that samples come from a single distribution.  Table 7 shows *t*-test *p* values with a Bonferroni correction. Based on these results, it can be inferred that, with statistical significance, *γ*_1_ configuration can be considered different from *β*_1_ and *α*_1_ configurations, based on a significance level of 0.05. Subsequently, Tukey HSD test results can be found in Table 7, to uphold that *γ*_1_ configuration is significantly different from the other configurations. Once the previous results were found, a higher performance for *γ*_1_ configuration is noticeable in the three left-most configurations shown in, e.g., Fertility (Figure 5), Glass (Figure 6), and Ionosphere (Figure 7), performance box plots.

### 4.2. Test of Designs Driven by Accuracy Error Fitness Function

Statistical analysis for configurations driven by accuracy error fitness function was performed with the same approach as in the previous subsection; Table 8 shows ANOVA, *t*-test, and Tukey HSD tests applied to such configurations. In this case, for designs driven by accuracy error fitness function, the pair-wise *t*-test show that there is not difference with statistical significance to reject the null hypothesis *H*_0_ for *α*_2_ and *γ*_2_ configurations; however, the *α*_2_ configuration requires a higher computational power to carry out the designing task due its search engine and its respective operators (crossover and mutation). The aforementioned issues are not presented for the *γ*_2_ configuration; besides its results show a similar accuracy results with lower dispersion, this can be noticed in the right-most configurations shown in, e.g., Fertility (Figure 5), Glass (Figure 6), and Ionosphere (Figure 7) performance box plots, and this behavior was consistently observed for all benchmark datasets. The Tukey HSD test shows that there is statistical difference for all configurations; this, along with the observed behavior in the previous box plots, confirms that *γ*_2_ configuration holds as the outperforming algorithm.

### 4.3. Test of All Configurations

An omnibus test was applied to the entire set of experiments considering both as independent variables, configurations and fitness functions. Two-way ANOVA test was applied to determine if varying both observed variables influences accuracy performance.  Table 9 contains such results, providing statistical certainty to reject the null hypothesis *H*_0_; in other words, the accuracy performance is affected by both variables. The *p* values lower than the significance level of 0.05 indicate that changing the optimization function (squared error and accuracy error) and the configuration does indeed affect the performance accuracy obtained by the generated topology.

Finally, pairwise *t*-test was applied to discern if, given two configurations, their performances are statistically similar. Considering *p* values in Table 9 and a significance value of 0.05, it can be inferred with statistically trustworthy that *γ*_2_ configuration generally outperforms other configurations.

## 5. Conclusions and Future Work

This paper presents a GE-based methodology to design partially connected ANNs for solving supervised classification problems; some interesting characteristics of the methodology are that it provides weighted topologies which allow us to avoid an explicit training and those topologies exhibit partial connectivity between input and hidden layers which may avoid redundancies and reduce the dimensionality of the input feature vectors. The proposed methodology (*γ*_2_) evolved from progressive improvements made to a base methodology (*α*_1_), which uses GE with GA as search engine and squared error as fitness function; improvements were made by changing neuron models which allowed us to generate SNNs (*β*_1_) instead of ANNs from the second ANN generation and by changing the search engine by using DE (*γ*_1_) instead of GA. All the aforementioned configurations were adapted to use another fitness function based on the accuracy error of generated ANNs, so-called *α*_2_, *β*_2_, and *γ*_2_.

In order to validate the achieved improvements, several statistics tests were applied. Each configuration was tested for twelve well-known benchmark datasets of supervised classification problems by performing 33 experiments for each dataset. Three types of statistical analysis were performed, and the first being applied to *α*_1_, *β*_1_, and *γ*_1_ configurations, which use squared error as the fitness function. In such analysis, *γ*_1_ configuration is shown to outperform the other configurations based on the statistical test and graphic box plots. The second analysis focused *α*_2_, *β*_2_, and *γ*_2_ configurations, which use the accuracy error as the fitness function; based on the Tukey HSD test, this analysis yielded a similar conclusion as from the first analysis, but with respect to *γ*_2_ configuration. The last analysis compared all configurations and showed statistical evidence to support that *γ*_2_ is a better configuration with competitive performances and lower dispersions for its designs.

Focusing in topology designs and performance results, evolutionary designs led to the formulation of solution topologies with fewer connections than those in equivalent fully connected topologies, hence reducing the complexity of the networks and achieving good classification performances. The topology simplification provided a good network design (i.e., design accuracy was competent), but it was desirable to get better generalization capability for unseen data in the test phase; some particular cases exhibited lower test accuracies, evidencing an improving opportunity.

Due to the flexibility of the context-free grammars employed in GE, another aspect of neural network topologies can be considered to cope with detected issues while preserving the enhancements accomplished. The design process may consider other traits, e.g., selection of the neural model and/or the search engine, specification of the model parameters, or even aggregation on the number of hidden layers to design SNNs for deep learning topologies. Moreover, additional types of topologies with structures other than layered networks can be explored to be designed, such as those of reservoir computing or central pattern generators. Furthermore, another kind of grammar-based genetic programming algorithms can be used to add semantic to the design process, such as Christiansen grammar evolution [67].

Finally, contemplating the fitness function as another relevant aspect to produce enhanced designs, considerations can also be made to it: to minimize the amount of processing units in the hidden layer or to consider another evaluation measurements to comply with other kinds of problems; features in the fitness function may be treated as weighted mono-objective fitness function or by using algorithms such as the nondominated sorting genetic algorithm (NSGA) [76] with fitness functions with multiple objectives.

## Figures and Tables

**Table 1:** Datasets employed for experimentation.

Dataset	Instances	Classes	Features
Balance Scale	625	3	4
Blood	748	2	4
Breast Cancer	683	2	9
Card	653	2	15
Diabetes	768	2	8
Fertility	100	2	9
Glass	214	6	9
Ionosphere	351	2	33
Iris Plant	150	3	4
Liver	345	2	6
Parkinson	195	2	22
Wine	178	3	13

**Table 2:** Configurations included in experimentation.

Configuration	Fitness function	Search engine
*α*_1_	Squared error	GA
*α*_2_	Accuracy error	GA
*β*_1_	Squared error	GA
*β*_2_	Accuracy error	GA
*γ*_1_	Squared error	DE
*γ*_2_	Accuracy error	DE

**Table 3:** Accuracy of design and testing on every configuration for Balance Scale, Blood, Breast Cancer, Card, Diabetes, and Fertility datasets.

Dataset	Configuration	Design accuracy	Test accuracy
Balance Scale	*α*_1_	0.7486 ± 0.0525	0.7219 ± 0.0629
	*α*_2_	0.8354 ± 0.0460	0.8077 ± 0.0582
	*β*_1_	0.7331 ± 0.0718	0.6944 ± 0.0752
	*β*_2_	0.8363 ± 0.0272	0.8078 ± 0.0427
	*γ*_1_	0.8528 ± 0.0197	0.8346 ± 0.0261
	*γ*_2_	**0.8960** **±** **0.0062**	**0.8647** **±** **0.0134**

Blood	*α*_1_	0.7711 ± 0.0112	0.7622 ± 0.0117
	*α*_2_	**0.8010** **±** **0.0135**	**0.7731** **±** **0.0168**
	*β*_1_	0.7747 ± 0.0110	0.7607 ± 0.0138
	*β*_2_	0.7863 ± 0.0162	0.7684 ± 0.0120
	*γ*_1_	0.7760 ± 0.0076	0.7618 ± 0.0088
	*γ*_2_	0.7957 ± 0.0145	0.7685 ± 0.0155

Breast Cancer	*α*_1_	0.9494 ± 0.0141	0.9418 ± 0.0238
	*α*_2_	**0.9781** **±** **0.0073**	**0.9585** **±** **0.0077**
	*β*_1_	0.9474 ± 0.0121	0.9405 ± 0.0151
	*β*_2_	0.9677 ± 0.0073	0.9432 ± 0.0142
	*γ*_1_	0.9574 ± 0.0111	0.9384 ± 0.0140
	*γ*_2_	0.9749 ± 0.0062	0.9478 ± 0.0117

Card	*α*_1_	0.8624 ± 0.0099	**0.8641** **±** **0.0140**
	*α*_2_	0.8779 ± 0.0160	0.8591 ± 0.0174
	*β*_1_	0.8561 ± 0.0502	0.8524 ± 0.0527
	*β*_2_	0.8814 ± 0.0137	0.8561 ± 0.0153
	*γ*_1_	0.8740 ± 0.0134	0.8596 ± 0.0197
	*γ*_2_	**0.8879** **±** **0.0120**	0.8535 ± 0.0166

Diabetes	*α*_1_	0.7506 ± 0.0231	0.7457 ± 0.0207
	*α*_2_	0.7843 ± 0.0156	0.7476 ± 0.0224
	*β*_1_	0.7570 ± 0.0151	0.7490 ± 0.0215
	*β*_2_	0.7780 ± 0.0143	**0.7477** **±** **0.0126**
	*γ*_1_	0.7810 ± 0.0153	0.7370 ± 0.0152
	*γ*_2_	**0.7902** **±** **0.0134**	0.7389 ± 0.0205

Fertility	*α*_1_	0.8988 ± 0.0256	0.8218 ± 0.0616
	*α*_2_	0.9297 ± 0.0204	0.8170 ± 0.0551
	*β*_1_	0.9255 ± 0.0243	0.8309 ± 0.0459
	*β*_2_	0.9182 ± 0.0222	0.8279 ± 0.0467
	*γ*_1_	**0.9455** **±** **0.0199**	**0.8479** **±** **0.0462**
	*γ*_2_	0.9370 ± 0.0131	0.8236 ± 0.0484

**Table 4:** Accuracy of design and testing on every configuration for Glass, Ionosphere, Iris Plant, Liver, Parkinson, and Wine datasets.

Dataset	Configuration	Design accuracy	Test accuracy
Glass	*α*_1_	0.2549 ± 0.1345	0.2404 ± 0.1433
	*α*_2_	0.6035 ± 0.0448	0.5374 ± 0.0688
	*β*_1_	0.4288 ± 0.0673	0.4002 ± 0.0590
	*β*_2_	0.6641 ± 0.0255	0.5947 ± 0.0436
	*γ*_1_	0.4895 ± 0.0574	0.4351 ± 0.0476
	*γ*_2_	**0.7126** ± **0.0190**	**0.6186** ± **0.0413**

Ionosphere	*α*_1_	0.8549 ± 0.0374	0.8374 ± 0.0295
	*α*_2_	0.9158 ± 0.0217	0.8669 ± 0.0267
	*β*_1_	0.8543 ± 0.0537	0.8137 ± 0.0708
	*β*_2_	0.9190 ± 0.0284	0.8724 ± 0.0241
	*γ*_1_	0.9351 ± 0.0182	0.8907 ± 0.0240
	*γ*_2_	**0.9616** ± **0.0113**	**0.9015** ± **0.0201**

Iris Plant	*α*_1_	0.8857 ± 0.1111	0.8663 ± 0.1269
	*α*_2_	0.9653 ± 0.0161	**0.9386** ± **0.0210**
	*β*_1_	0.9733 ± 0.0157	0.9382 ± 0.0217
	*β*_2_	0.9859 ± 0.0109	0.9362 ± 0.0163
	*γ*_1_	0.9794 ± 0.0123	0.9325 ± 0.0164
	*γ*_2_	**0.9923** ± **0.0074**	0.9358 ± 0.0261

Liver	*α*_1_	0.6820 ± 0.0406	0.6462 ± 0.0536
	*α*_2_	0.7352 ± 0.0245	0.6660 ± 0.0356
	*β*_1_	0.6834 ± 0.0481	0.6304 ± 0.0476
	*β*_2_	0.7461 ± 0.0224	0.6632 ± 0.0394
	*γ*_1_	0.7472 ± 0.0183	**0.6723** ± **0.0302**
	*γ*_2_	**0.7636** ± **0.0196**	0.6612 ± 0.0295

Parkinson	*α*_1_	0.8719 ± 0.0395	0.8281 ± 0.0568
	*α*_2_	0.9080 ± 0.0159	**0.8596** ± **0.0285**
	*β*_1_	0.8563 ± 0.0264	0.8033 ± 0.0519
	*β*_2_	0.8953 ± 0.0205	0.8503 ± 0.0353
	*γ*_1_	0.9025 ± 0.0266	0.8380 ± 0.0387
	*γ*_2_	**0.9200** ± **0.0172**	0.8494 ± 0.0377

Wine	*α*_1_	0.6881 ± 0.1549	0.6415 ± 0.1551
	*α*_2_	0.9063 ± 0.0441	0.8384 ± 0.0606
	*β*_1_	0.7375 ± 0.1126	0.6816 ± 0.1098
	*β*_2_	0.8895 ± 0.0641	0.7855 ± 0.0686
	*γ*_1_	0.9318 ± 0.0285	0.8620 ± 0.0491
	*γ*_2_	**0.9638** ± **0.0164**	**0.8684** ± **0.0458**

**Table 5:** Topology characteristics for every dataset on configurations *α*_1_, *β*_1_, and *γ*_1_.

Configuration	Dataset	Average number of features employed	Rate of used features	Average number of hidden units	Average number of synapses
*α*_1_	Balance Scale	2.97 ± 0.67	0.74	1.58 ± 0.85	9.58 ± 2.89
	Blood	2.30 ± 0.76	0.58	1.85 ± 0.99	7.64 ± 2.24
	Breast Cancer	2.52 ± 0.99	0.28	2.03 ± 1.09	8.79 ± 2.04
	Card	2.52 ± 0.93	0.17	2.09 ± 0.93	8.67 ± 2.22
	Diabetes	2.09 ± 0.90	0.26	1.97 ± 1.11	7.09 ± 1.91
	Fertility	3.03 ± 0.97	0.34	2.30 ± 1.29	9.24 ± 2.87
	Glass	2.21 ± 1.32	0.25	1.48 ± 0.93	11.73 ± 3.77
	Ionosphere	2.24 ± 1.05	0.07	2.21 ± 1.30	7.61 ± 2.81
	Iris Plant	1.30 ± 0.52	0.33	1.70 ± 1.00	8.18 ± 2.35
	Liver	2.48 ± 0.70	0.41	1.88 ± 0.84	6.64 ± 1.79
	Parkinson	2.27 ± 1.38	0.10	1.85 ± 1.50	8.00 ± 4.65
	Wine	2.48 ± 1.23	0.19	1.97 ± 1.75	9.73 ± 4.48

*β*_1_	Balance Scale	3.52 ± 0.56	0.88	4.09 ± 1.58	12.36 ± 4.32
	Blood	3.12 ± 0.69	0.78	4.76 ± 2.55	12.03 ± 5.77
	Breast Cancer	6.12 ± 1.47	0.68	4.03 ± 1.85	14.24 ± 5.46
	Card	5.79 ± 1.95	0.39	4.39 ± 2.09	12.85 ± 5.47
	Diabetes	3.39 ± 0.95	0.42	3.55 ± 1.71	09.36 ± 4.00
	Fertility	5.58 ± 1.74	0.62	4.09 ± 2.22	12.39 ± 6.02
	Glass	5.70 ± 1.62	0.63	3.03 ± 1.82	11.88 ± 6.30
	Ionosphere	5.52 ± 2.24	0.17	3.55 ± 2.05	12.15 ± 6.68
	Iris Plant	3.39 ± 0.89	0.85	4.73 ± 2.60	13.27 ± 6.93
	Liver	3.94 ± 1.07	0.66	4.06 ± 1.91	11.03 ± 5.32
	Parkinson	5.12 ± 1.77	0.23	4.36 ± 2.45	11.48 ± 5.66
	Wine	6.15 ± 1.88	0.47	3.82 ± 1.49	13.03 ± 4.93

*γ*_1_	Balance Scale	4.00 ± 0.00	1.00	4.03 ± 1.64	12.79 ± 3.75
	Blood	3.79 ± 0.48	0.95	4.70 ± 1.71	13.42 ± 4.23
	Breast Cancer	6.70 ± 1.38	0.74	5.39 ± 2.39	16.30 ± 5.93
	Card	8.76 ± 2.85	0.58	5.12 ± 2.04	18.03 ± 8.12
	Diabetes	6.24 ± 1.33	0.78	5.67 ± 3.19	17.76 ± 9.36
	Fertility	7.24 ± 1.18	0.80	4.79 ± 2.42	16.79 ± 6.20
	Glass	6.79 ± 1.32	0.75	4.97 ± 2.18	16.24 ± 5.85
	Ionosphere	11.64 ± 3.31	0.35	4.45 ± 1.86	18.03 ± 6.07
	Iris Plant	3.91 ± 0.29	0.98	5.79 ± 2.79	15.73 ± 6.89
	Liver	5.03 ± 1.03	0.84	4.64 ± 2.36	14.39 ± 6.49
	Parkinson	8.91 ± 3.41	0.40	4.18 ± 2.37	15.06 ± 7.58
	Wine	8.64 ± 1.92	0.66	4.97 ± 1.62	16.97 ± 5.33

**Table 6:** Topology characteristics for every dataset on configurations *α*_2_, *β*_2_, and *γ*_2_.

Configuration	Dataset	Average number of features employed	Rate of used features	Average number of hidden units	Average number of synapses
*α*_2_	Balance Scale	3.73 ± 0.45	0.93	2.06 ± 1.23	11.48 ± 3.47
	Blood	3.33 ± 0.77	0.83	2.52 ± 1.28	11.64 ± 3.95
	Breast Cancer	3.85 ± 1.10	0.43	2.06 ± 1.04	10.82 ± 3.93
	Card	5.39 ± 2.20	0.36	2.76 ± 1.54	14.42 ± 6.27
	Diabetes	3.21 ± 0.95	0.40	2.33 ± 1.22	10.64 ± 3.56
	Fertility	5.30 ± 1.59	0.59	2.94 ± 2.01	15.39 ± 7.72
	Glass	2.79 ± 1.22	0.31	1.88 ± 0.91	13.91 ± 3.70
	Ionosphere	4.45 ± 1.67	0.13	3.55 ± 1.42	14.18 ± 4.36
	Iris Plant	1.76 ± 1.05	0.44	2.00 ± 1.67	11.27 ± 6.49
	Liver	3.33 ± 0.94	0.56	1.67 ± 0.84	09.18 ± 3.02
	Parkinson	3.67 ± 2.22	0.17	1.91 ± 1.03	10.09 ± 4.43
	Wine	3.15 ± 1.52	0.24	2.67 ± 1.49	12.30 ± 5.26

*β*_2_	Balance Scale	3.88 ± 0.33	0.97	3.39 ± 1.92	09.88 ± 4.16
	Blood	3.21 ± 0.69	0.80	4.21 ± 3.50	11.55 ± 9.49
	Breast Cancer	5.76 ± 1.63	0.64	3.97 ± 2.41	11.94 ± 6.09
	Card	6.94 ± 2.24	0.46	4.55 ± 2.85	14.00 ± 7.21
	Diabetes	4.06 ± 1.23	0.51	3.91 ± 2.11	10.85 ± 5.65
	Fertility	5.30 ± 2.11	0.59	3.39 ± 2.01	10.36 ± 5.64
	Glass	5.18 ± 1.45	0.58	3.73 ± 1.66	11.21 ± 4.44
	Ionosphere	6.48 ± 2.27	0.20	4.30 ± 2.67	12.94 ± 6.33
	Iris Plant	3.24 ± 0.74	0.81	4.09 ± 2.50	10.61 ± 6.30
	Liver	4.27 ± 1.21	0.71	3.52 ± 1.78	10.52 ± 4.39
	Parkinson	5.39 ± 2.44	0.25	3.09 ± 1.99	09.64 ± 5.51
	Wine	5.85 ± 1.73	0.45	4.24 ± 2.22	12.85 ± 5.21

*γ*_2_	Balance Scale	4.00 ± 0.00	1.00	3.15 ± 2.12	10.55 ± 5.78
	Blood	3.67 ± 0.59	0.92	4.58 ± 2.56	12.79 ± 5.86
	Breast Cancer	7.30 ± 1.27	0.81	4.61 ± 1.82	15.85 ± 5.21
	Card	8.58 ± 2.45	0.57	4.00 ± 2.47	15.09 ± 7.10
	Diabetes	6.06 ± 1.41	0.76	4.24 ± 1.78	13.76 ± 4.96
	Fertility	6.73 ± 1.33	0.75	4.06 ± 2.73	14.21 ± 6.65
	Glass	6.70 ± 1.17	0.74	4.91 ± 1.60	15.06 ± 4.26
	Ionosphere	11.64 ± 4.14	0.35	3.88 ± 2.20	16.88 ± 7.10
	Iris Plant	3.61 ± 0.69	0.90	3.82 ± 2.18	10.97 ± 5.42
	Liver	4.91 ± 0.90	0.82	3.21 ± 1.53	10.45 ± 4.11
	Parkinson	7.97 ± 2.47	0.36	3.39 ± 1.50	12.36 ± 4.20
	Wine	8.15 ± 1.96	0.63	4.70 ± 1.93	16.12 ± 5.57

**Table 7:** Two-way ANOVA *F*, pairwise *t*-test, and Tukey HSD test with Bonferroni correction for squared error configurations.

ANOVA	Df	Sum Sq	Mean Sq	*F* value	Pr(>*F*)
Configuration	2	0.7109	0.35547	73.217	**<2.2e-16**
Dataset	11	25.3929	2.30845	475.4710	**<2.2e-16**
Residuals	1174	5.6999	0.00486		

*t*-Tests	*α*_1_		*β*_1_		

*β*_1_	**0.5936**		—		
*γ*_1_	**1.9e-6**		**0.0006**		

Tukey HSD		diff	lwr	upr	p adj

*β*_1_ vs. *γ*_1_		0.014	0.0032	0.0544	**0.00**
*α*_1_ vs. *β*_1_		0.014	0.0030	0.026	0.0078
*α*_1_ vs. *γ*_1_		0.057	0.0460	0.0693	**0.00**

**Table 8:** Two-way ANOVA *F*, pairwise *t*-test, and Tukey HSD test with Bonferroni correction for accuracy error configurations.

ANOVA	Df	Sum Sq	Mean Sq	F value	Pr(>F)
Configuration	2	0.0769	0.0384	26.319	**6.58*e*** − **12**
Dataset	11	12.3300	1.1210	767.4670	**<2.2*e*** − **16**
Residuals	1174	1.7160	0.0014		

*t*-Tests	*α*_2_		*β*_2_		

*β*_2_	**1.0000**		—		
γ_2_	**0.1030**		**1.3*e*** − **3**		

Tukey HSD		diff	lwr	upr	p adj

*β*_2_ vs. *γ*_2_		0.0170	0.0110	0.0240	**0.0000**
*α*_2_ vs. *β*_2_		−0.0010	−0.0070	0.0050	0.8830
*α*_2_ vs. *γ*_2_		0.0100	0.0100	0.0220	**0.0001**

**Table 9:** Two-way ANOVA *F* and pairwise *t*-test with Bonferroni correction tests for all configurations.

ANOVA	Df	Sum Sq	Mean Sq	F value	Pr(>F)
Fitness function	1	1.1220	1.1200	58.9180	**2.36*e*** − **14**
Configuration	4	0.7880	0.1960	10.3400	**2.663*e*** − **08**
Residuals	2370	45.1490	0.0190		

*t*-Tests	*α*_1_	*α*_2_	*β*_1_	*β*_2_	*γ*_1_

*α*_2_	0.0001	—	—	—	—
*β*_1_	0.0100	0.00	—	—	—
*β*_2_	0.0001	0.9900	0.0010	—	—
*γ*_1_	0.0001	0.8900	0.0010	0.9680	—
*γ*_2_	0.0000	0.0040	0.0000	0.0010	4.8*e* − 5

## Data Availability

The supervised classification dataset benchmarks used to support the findings of this study have been taken from the UCI Machine Learning Repository of the University of California, Irvine (http://archive.ics.uci.edu/ml/datasets.html).

## Supplementary Materials

Supplementary Materials Examples of the best results obtained for SNNs are shown; each example contains the benchmark dataset, used configuration, accuracies of design and test phases, the generated word, and the network topology.
