# Supplementary material for: Evolutionary Spiking Neural Networks for Solving Supervised Classification Problems
Source: Comput Intell Neurosci. 2019 Mar 28;2019:4182639. doi: 10.1155/2019/4182639 (PMC6458934; doi:10.1155/2019/4182639)

Appendix A. Examples of the best networks obtained

Dataset: Parkinson
Configuration: β_2
Acurracy of design: 0.8762
Accuracy of test: 0.9183
Corresponding word:

```
0@+006.19,05.09(17@-905.03,04.46(o@+919.47,01.06{
17@-865.36,01.08(16@-785.57,03.96(o@+241.11,06.99{
4@+677.06,00.44(19@-935.31,02.36(o@+054.47,00.29{
```

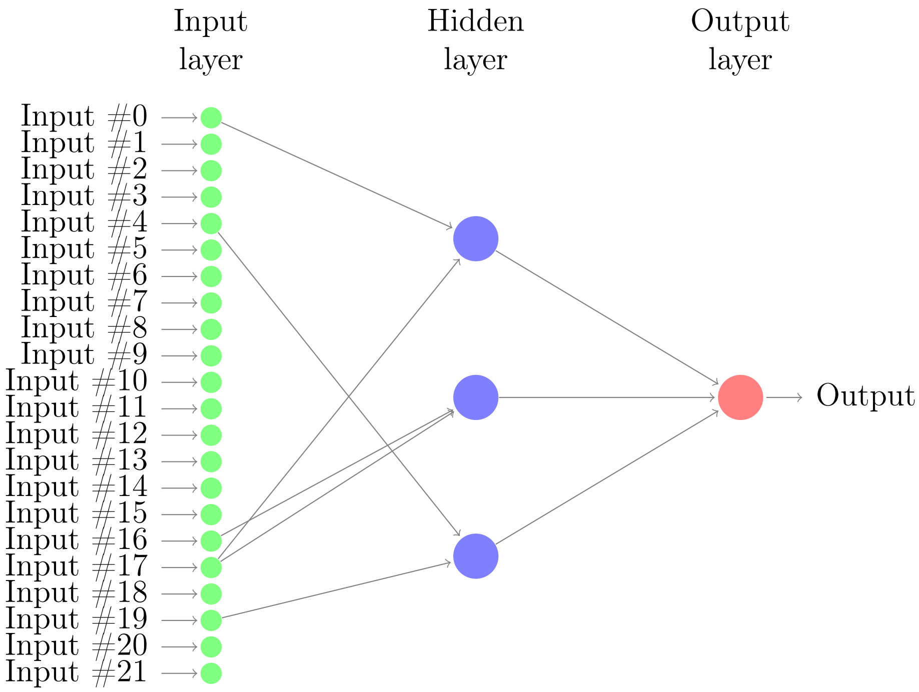

Dataset: Iris Plant
Configuration: γ_2
Acurracy of design: 1.0000
Accuracy of test: 0.9600
Corresponding word:

```
2@+626.94,08.32(1@-829.47,07.16(0@-057.27,05.57(o@+428.91,00.63{
```

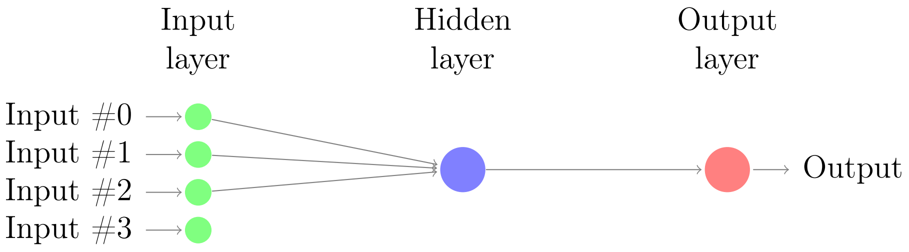

Dataset: Fertility
Configuration: γ_2
Acurracy of design: 0.92
Accuracy of test: 0.92
Corresponding word:

```
5@+771.61,11.57(1@-322.51,11.54(o@+109.55,03.11{
3@-133.67,03.16(1@+085.76,07.24(8@+279.02,07.03(4@+534.86,00.63(o@+199.01,02.23{
7@-703.42,16.37(o@-735.66,10.81{
4@-018.37,12.29(o@-995.42,13.94{
0@-016.79,04.69(o@+822.55,09.71{
```

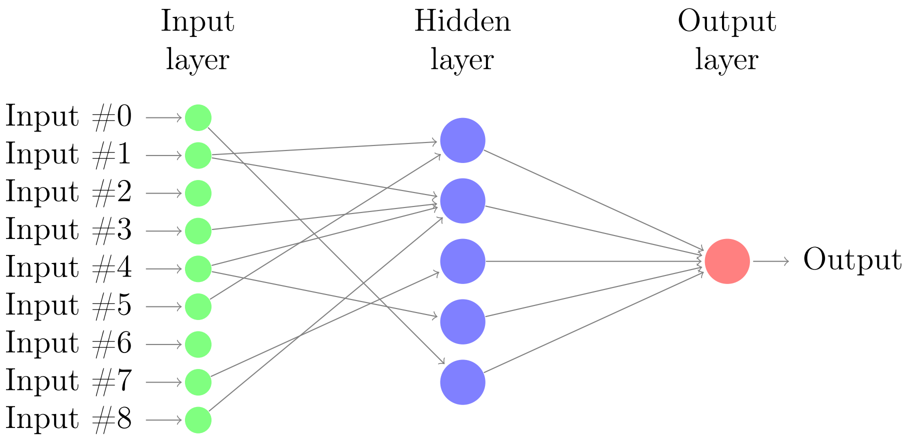

Dataset: Wine
Configuration: γ_2
Acurracy of design: 1.0000
Accuracy of test: 0.8333
Corresponding word:

```
9@+378.67,07.72(2@+493.12,05.28(o@+029.66,03.64{
11@+445.42,15.37(10@+485.27,13.04(o@-245.21,13.84{
11@+389.39,10.02(o@-846.47,00.89{
6@-656.89,05.94(8@+033.62,01.37(0@-382.17,06.39(o@+636.03,01.53{
12@-006.61,04.22(2@+139.17,08.21(o@+423.19,03.45{
```

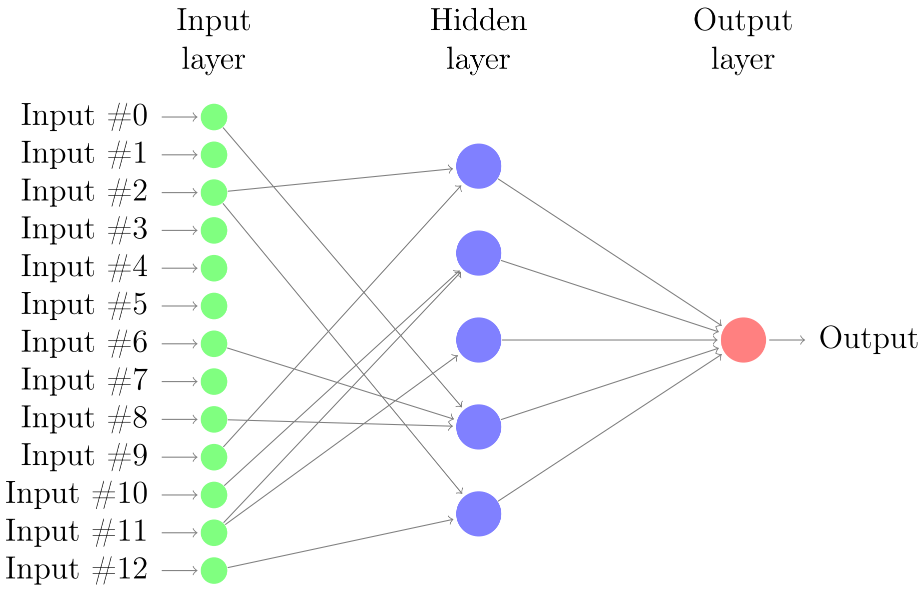

Dataset: Balance Scale
Configuration: γ_2
Acurracy of design: 0.9071
Accuracy of test: 0.8498
Corresponding word:

```
2@+495.07,05.92(0@-508.06,05.63(3@+426.12,05.21(1@-616.39,06.95(o@+695.78,03.23{
0@+976.28,18.74(o@-807.75,08.41{
2@-944.25,17.81(o@+170.28,07.29{
```

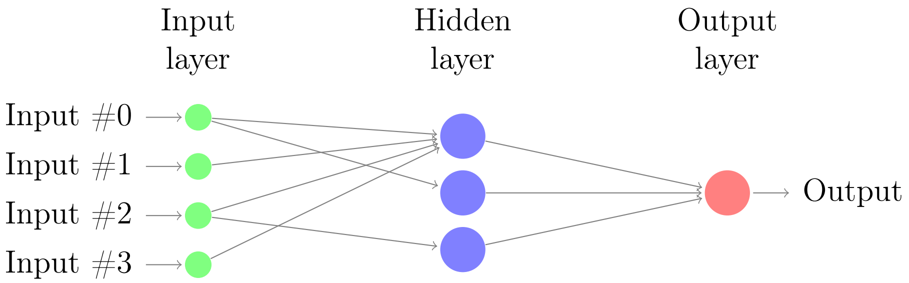

Supplement: Supplementary Materials — Examples of the best results obtained for SNNs are shown; each example contains the benchmark dataset, used configuration, accuracies of design and test phases, the generated word, and the network topology. [file 4182639.f1.pdf]
